# Where Failure Is Not an Option –Personalized Medicine in Astronauts

**DOI:** 10.1371/journal.pone.0140764

**Published:** 2015-10-21

**Authors:** Julia C. Stingl, Susanne Welker, Gunther Hartmann, Volker Damann, Ruppert Gerzer

**Affiliations:** 1 Research Division, Federal Institute for Drugs and Medical Devices, Bonn, Germany; 2 Centre for Translational Medicine, University Bonn Medical Faculty, Bonn, Germany; 3 Institute for clinical chemistry and clinical pharmacology, University of Bonn, Bonn, Germany; 4 Space Medicine Office, European Space Agency, Cologne, Germany; 5 Institute of Aerospace Medicine, German Aerospace Center, Cologne, Germany; Tel Aviv University, Israel, ISRAEL

## Abstract

Drug safety and efficacy are highly variable among patients. Most patients will experience the desired drug effect, but some may suffer from adverse drug reactions or gain no benefit. Pharmacogenetic testing serves as a pre-treatment diagnostic option in situations where failure or adverse events should be avoided at all costs. One such situation is human space flight. On the international space station (ISS), a list of drugs is available to cover typical emergency settings, as well as the long-term treatment of common conditions for the use in self-medicating common ailments developing over a definite period. Here, we scrutinized the list of the 78 drugs permanently available at the ISS (year 2014) to determine the extent to which their metabolism may be affected by genetic polymorphisms, potentially requiring genotype-specific dosing or choice of an alternative drug. The purpose of this analysis was to estimate the potential benefit of pharmacogenetic diagnostics in astronauts to prevent therapy failure or side effects.

## Introduction

Drug safety and efficacy are highly variable among patients. Most patients will experience the desired drug effect, but some may suffer from adverse drug reactions or gain no benefit. For this reason, treatment strategies in which individual patient factors are taken into account in the choice and dosing of a drug have been promoted as superior to the “one-drug-fits-all” approach, especially in situations where drug failure or toxicity is not tolerated.

This state of affairs may reflect the way evolution has addressed the problem of designing genetic codes for enzymes that should be able to metabolize an unpredictably large and varying range of exogenous substances. This wide net is cast by a whole family of related enzymes, whose genetic specification reacts to evolutionary pressure by producing variants that are more numerous in ethnic groups which have been historically exposed by exceptional rates of xenobiotics through life style or diet. Furthermore, these enzymes can cope with diverse substrates. Substrates, in turn, are often metabolized by several different enzymes. This setting appears to be particularly conducive to the existence of genetic variants in what are now known as drug metabolizing enzymes which are the main known source of pharmacogenetic variability in drug exposure [1, 2].

Pharmacogenetic-related variation in drug exposure (plasma concentrations, elimination rates) is substantial, corresponding to differences in dosing in the rage of two-to tenfold [3]. Thus, especially when the therapeutic range is relatively narrow, these differences in drug exposure can have an enormous impact on drug response or adverse drug reactions [4].

When large pharmacokinetic effects are accompanied by narrow therapeutic ranges and the ensuing risks of toxicity or therapeutic failure, the judicious use of genotypic information to adjust dosing is in order. Evidence-based guidelines have been issued and are further being developed for gene-drug pairs where strong supporting evidence exist that genetic variations clearly affect drug efficacy or the risk of adverse reactions [5–9].

Pharmacogenetic testing may also be viewed as a guiding diagnostic instrument in order to prevent therapeutic failure or adverse drug reactions. Routine pre-treatment genetic testing in the general population is often criticized because of its low cost-effectiveness. However, utility and effectiveness of pharmacogenetic testing may be judged differently in situations where failure or adverse events should be avoided at all costs.

One such situation is human space flight. On the international space station (ISS), available drugs cover typical emergency settings, as well as the long-term treatment of common conditions. Thus, the drugs that are available on the ISS can be regarded as representative of those required in a typical sample of essentially healthy individuals, faced with the task of self-medicating common ailments developing over a definite period. Most frequent indications (according to ATC codes of the drugs) are infections, pain and inflammation, nausea, sleeping disturbances, allergies followed by gastrointestinal disorders, hypertension and psychic conditions. The pharmacogenetics of the ISS pharmacy may therefore be viewed as a compendium of the effects of genetic variability on medication in human beings who, in an international setting, may be sampled from fairly different ancestries.

## Results

Here, we scrutinized the list of the 78 drugs permanently available at the ISS (year 2014) to determine the extent to which their elimination may be affected by genetic polymorphisms, potentially requiring genotype-specific dosing or choice of an alternative drug. The purpose of this analysis was to estimate the potential benefit of pharmacogenetic diagnostics in astronauts to prevent therapy failure or side effects. In Table 1, drugs that are affected by genetic polymorphisms are presented including information on the metabolism and transport of these drugs obtained from the drug labels approved by the US Food and Drug Administration (FDA) or/and the European Medicines Agency (EMA) or from the European Public Assessment Reports (EPARs).

**Table 1:** Polymorphic CYP substrates within the ISS drug list: Information about involvement of polymorphic drug metabolism from the drug labels or from evidence-based pharmacogenetic guidelines. Among the drugs listed in the ISS repository, specific warnings related to pharmacokinetic effects have been identified in the label section of 14 drugs. For six drugs on the list, specific therapy modifications (alternative drugs) or dosing adjustments are proposed in existing evidence-based guidelines.

CYP2D6 substrates on ISS drug list	Indication	Information about polymorphic enzymes in the drug label	Dosing Guidelines: CPIC/ GWPG	References	Level of evidence*
Metoprolol	Heart failure, hypertension	FDA: warnings about pharmacogenetics and drug interactions	PM: 75% UM: up to 250%	[10, 11]	3
Diphenhydramine	Vomiting, allergic rhinitis	Warning about drug interactions with drugs metabolized by CYP2D6		[12]	3
Cetirizine	Vomiting, allergic rhinitis	Information about drug metabolism via CYP2D6		[13]	1
Loratadine	Vomiting, allergic rhinitis, urticaria	Information about drug metabolism via CYP2D6		[14]	1
Meclizine	Vomiting, allergic rhinitis	Information about drug metabolism via CYP2D6		[15]	1
Ondansetron	vomiting	Information about drug metabolism via CYP2D6		[16]	3
Promethazine	Rhinitis, urticarial, Sedation, vomiting	Information about drug metabolism via CYP2D6		[17]	3
Tamsulosin	Prostate hyperplasia	Information about drug metabolism, high exposure in PM as compared to EM		[18]	2
Acetaminophen	Pain, fever	Warning about interaction potential with CYP2D6 substrates		[19]	1
Hydrocodone	Pain	CYP2D6 involved in activation; PMs less efficacy		[20]	1
Venlafaxine	Depression	Metabolism of venlafaxine to the active metabolite, total active moiety not affected by polymorphism	80% in PMs 170% in UMs or select an alternative drug, Cardiotoxic risk higher in PMs	[21, 22]	3
Aripiprazole	Psychosis	Dose recommendations in FDA label, and interaction warning	Reduce dose in PMs to 67% UMs no recommendation	[23]	2
CYP2C19 substrates					
Diazepam	Sleep disturbances	Information about drug metabolism and interaction via CYP2C19		[24]	2
Sertraline	Depression	Information about drug metabolism via CYP2C19	Reduce PM dose to 50% UMs no recommendation	[6]	2
Omeprazole	Reflux	Drug interactions	UM dose 100–200% increased	[25]	3
CYP2C9 substrates					
Ibuprofen	Pain, Fever	CYP2C9 and CYP2C8 involved in metabolism	*CYP2C8 and 9 combined genotype involved in GI bleeding side effects*	[26]	3
Phenytoin	Epilepsia, seizures	PMs: enhanced risk of toxicity	PMs: 50%, higher risk for skin toxicity; IMs: 75% of dose	[27]	3
Ketamine	Anesthesia, pain	Minor enzyme involved in metabolism		[28]	1
Acetylsalicylic acid	Pain, fever, cardiovascular	Minor enzyme, Drug interactions	CYP2C9 PM higher risk for urticaria	[29]	1
Sulfamethoxazole	Antibiotic	Information about m via CYP2C9	Risk of hemolysis in Glucose 6 phosphatase dehydrogenase deficiency	[30]	1
Loperamide	Diarrhea	Interaction warning		[31]	1
CYP1A2					
Melatonin	Daytime sleep, insomnia	Metabolism, Interactions		[32]	3
Caffeine	Sleepiness	Metabolism, Interactions		[33]	3
Lidocaine	Anaesthetic	Interactions		[34]	3

* Level of evidence: 1: in vitro data only, 2: in vivo pk data, 3: clinical data on efficacy and/or side effects

### ISS drugs affected by pharmacogenetic polymorphisms (Table 1)

**Fig 1:**
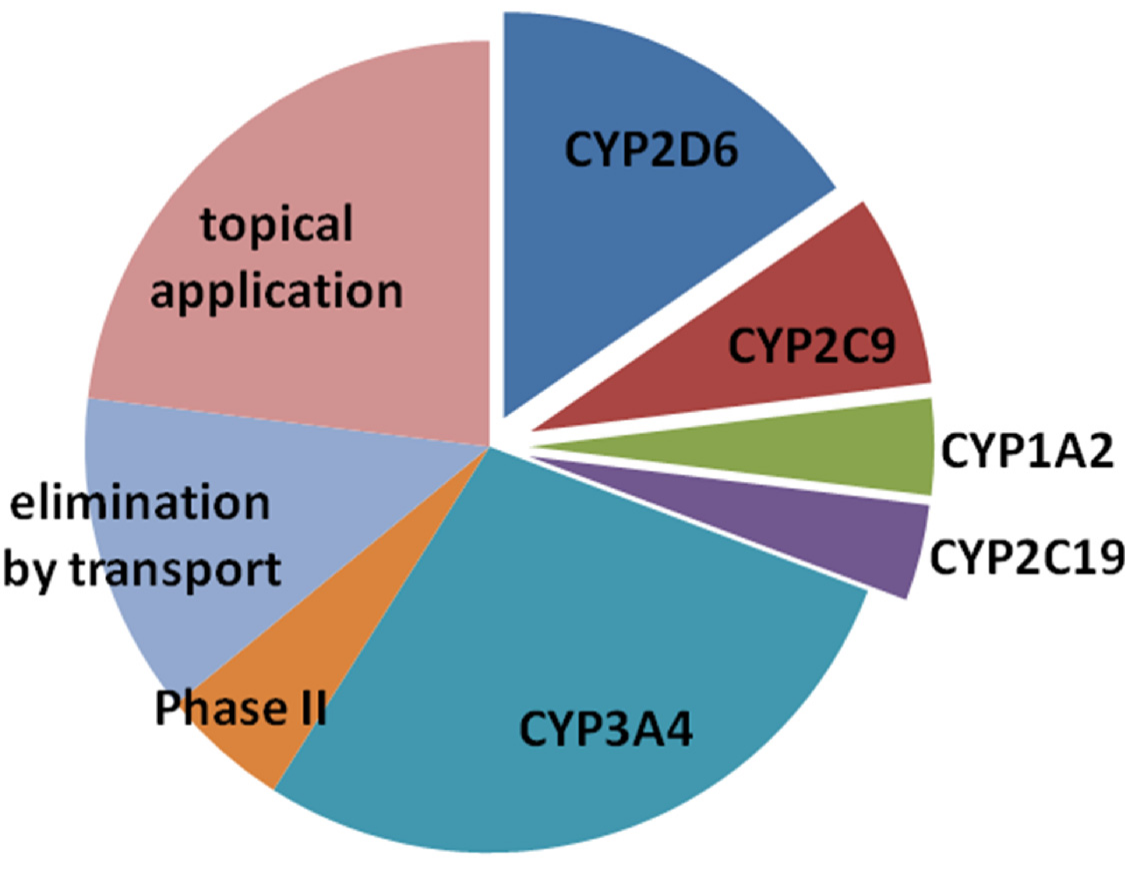
The main enzymes or transporters involved in elimination of the drugs listed in the ISS drug repository. The number of drugs metabolized mainly by the respected enzyme or transporter corresponds to the size of the circle partitions. The retracted slices of the circle are enzymes with known genetic polymorphisms and empirical data for the formulation of therapeutic recommendations or dose adjustments.

The ISS repository contains 78 drugs. Of these, 77% (n = 60) are applied systemically (orally or intravenously), while 23% (n = 18) require topical application. In the former group of drugs, where bioavailability is systemic, 72% (n = 43) are mainly eliminated by drug metabolism, 17% (n = 10) are mainly eliminated by known drug transporters, and 12% (n = 7) are eliminated unchanged or with unknown drug transporters. Our analysis revealed that in 24 (31%) of all drugs in the ISS repository (n = 78), metabolism is to a major extent affected by polymorphic metabolizing enzymes. Most frequently, the cytochrome P450 enzyme CYP2D6 was involved, followed by CYP2C19, CYP2C9 and CYP1A2. In the cases where elimination was mainly mediated by drug transport, the transporters P-glykoprotein (n = 4), PEPT1 (n = 2), OCT1 (n = 2), MRP1 (n = 1) and MCT (n = 1) were involved. However, there is not enough evidence at present on how genetic polymorphisms in these transporters affect drug exposure to formulate therapeutic recommendations or dose adjustments. In Fig 1, an overview on the main enzymes or transporters involved in elimination of the drugs listed in the ISS drug repository is given.

### Drugs metabolized by CYP2D6

Metabolizing about 20% of all drugs [35], CYP2D6 is a highly polymorphic enzyme that is expressed not only in hepatocytes but also in neurons in many regions in the brain [36]. CYP2D6 affects n = 11 (14%) of the ISS-listed drugs: 2 antipsychotics, one antidepressant, three antihistaminergic, 2 analgesics, and one betablocker, one hormone agonist and one antiemetic. All these drugs are active in the central nervous system, either because this latter constitutes their primary target, or because of notable side effects.

There are large interethnic differences in CYP2D6 drug metabolizer status. Large-sample studies in Caucasians found 7% poor metabolizers (individuals without any enzyme activity, PMs) and 3% ultrarapid metabolizers (with manifold enhancement of enzyme activity, UMs). However, the prevalence of UMs rises to about 10% in Spaniards, and up to 30% in Arabian countries [35]. In Asian populations, the polymorphism is less strong, with almost no true PMs or UMs but high frequencies of intermediate metabolizers [37]. Worldwide, more than 70 alleles have been identified coding for decreased, absent or increased enzyme activity (http://www.cypalleles.ki.se/). Since astronauts usually work in global and international teams, this group of potential drug consumers will represent most of the ethnic groups studied with regards to polymorphic drug metabolism.

The activity of the enzyme can be well predicted from allele composition. The allelic genotypes of *CYP2D6* can be directly translated into an activity score for this enzyme that ranges on seven activity levels between 0 and 3 times the activity level of the wild type [38]. These differences in enzyme activity lead to 4–10 fold differences in drug clearance of drugs that are metabolized by CYP2D6 [9].

One commonly used drug that is on the ISS list for acute or chronic pain treatment is hydrocodone. Hydrocodone, and to a similar extent codeine are converted to (hydro-)morphine via CYP2D6. After several cases of severe or fatal intoxication with codeine in children, the FDA and the EMA restricted the use in children and vulnerable patients and issued a warning about the issue that variable drug metabolism capacity may lead to dangerous intoxication with morphine. The hydrocodone drug label contains warning about the activation of the drug to hydromorphine by CYP2D6. Thus, in the situation in space, hydrocodone should be avoided in astronauts genotyped as CYP2D6 ultrarapid metabolizers because of a higher risk for hydromorphine induced respiratory depression. In the case of being a CYP2D6 poor metabolizer, hydrocodone may lack efficacy because this compound is 30-fold more potent than hydrocodone [20].

Another drug from the ISS list that is a CYP2D6 substrate is one of the most often-prescribed beta-blockers: metoprolol. Here, the CYP2D6 genetic polymorphism plays a well-known role in the pharmacokinetics of metoprolol. Not only metoprolol plasma concentrations but also effects on heart rate are stronger in CYP2D6 PMs and IMs compared to EMs [39]. Since about 30% of the population (Caucasian but as well Asian) are PMs or IMs, the intake of metoprolol in these individuals may lead to a higher risk of symptoms of bradycardia, and due to the higher drug levels to a reduced cardioselectivity with peripheral beta receptor inhibition related side effects. On the other side of the spectrum, CYP2D6 ultrarapid metabolizers may have about twofold higher clearances of metoprolol compared to EMs [40], and reduction of exercise-induced heart rate in UMs is only about half of that observed in EMs. Warnings about the effects of pharmacogenetic variants and drug interactions are given in the metoprolol drug label. The current dosing guideline of the Dutch Pharmacogenetic working party (GPWG) recommends a reduction of the metoprolol dose in CYP2D6 PMs (to 75% of the standard dose) and in increase in UMs (up to 250%).

For affective symptoms, depressive episodes or anxiety, the ISS drug list includes the antidepressant drugs venlafaxine and sertraline. Venlafaxine is metabolized to its active O-desmethyl-metabolite by CYP2D6 [41–43]. A higher risk for cardiotoxic events and other adverse drug effects may exist in individuals lacking CYP2D6 activity [21]. Cases of severe arrhythmias have been reported in four patients treated with venlafaxine who all were CYP2D6 PMs [22]. The GPWG guidelines recommend venlafaxine dosage adjustments of 80% of the standard dose in PMs and 170% in UMs.

There are several antihistaminic drugs on the ISS list that are suited for the treatment of nausea, kinetosis, allergic rhinitis and sleep disturbances: diphenhydramine, meclizine, cetirizine, and loratadine. According to the literature, these compounds are partly metabolized by the polymorphic CYP2D6 enzyme. Because these are old drugs, no patient data but only in vitro studies are available [12–14] [15] [17]. It seems rational to assume that PMs will have higher drug exposure at normal doses than EMs.

### Drugs metabolized by CYP2C19

CYP2C19 is a polymorphic enzyme mainly expressed in the liver and intestine. It is involved in the metabolism of a relatively small portion of all drugs, but in these drugs its polymorphism is of clinical relevance. This is the case for some benzodiazepines, proton pump inhibitors, several antidepressants, and the activation of the antiplatelet drug clopidogrel [3]. Several alleles lead to complete deficiency of the enzyme. The poor metabolizer phenotype is detected in only about 2% of Caucasians, but up to 20% in Asian populations [44]. The *17 allele leads to ultrarapid metabolism and is detected in 17% of Caucasians and less than 4% in Asians [45, 46].

The ISS drug list contains omeprazole, a Proton pump inhibitor (PPI), which is commonly used in gastroduodenal reflux disease. It undergoes extensive presystemic biotransformation in the liver with the involvement of genetically polymorphic CYP2C19 [47, 48]. Treatment of gastroesophageal reflux disease (GERD) demands long-term application of PPIs. Two analyses in Japanese patients showed that the healing rate of GERD was 20–40% higher in CYP2C19 PMs or IMs compared to EMs, and that PMs even benefit in the prevention of relapse of GERD [49–51]. In summary, according to the results of numerous clinical studies, the CYP2C19 polymorphism is an important factor affecting the pharmacokinetics of most PPIs. Therefore, especially in Asian individuals, which are characterized by a high prevalence of defect CYP2C19 alleles, genotyping for CYP2C19 may be indicated. The natural consequence of this approach would be higher dosages in extensive and ultrarapid metabolizers to obtain high enough efficacy in acid blocking effects.

For sleeping medication, the ISS list contains benzodiazepines, benzodiazepine receptor agonists and the abovementioned antihistaminergic substances. CYP2C19 PMs may have higher plasma concentrations of diazepam and zolpidem [24]; [52]. In contrast, the also ISS-listed lorazepam and the receptor agonists zaleplon are not metabolized by polymorphic enzymes [53, 54].

CYP2C19 affects the metabolism of the antidepressant sertraline. Poor metabolizers of CYP2C19 should be treated at reduced dosages (50% reduction) in order to avoid drug toxicity.

### Drugs metabolized by CYP2C9

CYP2C9 is primarily expressed in the liver, and contributes to metabolism of up to 15%-20% of all drugs [55]. Mainly two alleles (designated as *2 and *3) contribute to variations in enzyme activity. Homozygous carriers of the low activity variants show decreased but not complete enzyme deficiency. There are also interethnic differences in the allele frequencies, as the relatively higher rates of the *3 low activity allele (around 10%) in Southern Europeans are not found in Asians [56]. The nonsteroidal anti-inflammatory drugs (NSAIDs), which are represented in the ISS drug list by 3 compounds, are known to be metabolized via CYP2C9. Significant inter-genotypic differences were reported in the pharmacokinetics of ibuprofen, translating into dose recommendations with reductions to 60% of the normal dose in CYP2C9*3/*3 carriers [55].

Phenytoin, an antiepileptic drug on the ISS list, has a small therapeutic range and is used in the treatment of seizures and epilepsia. 50% dose reductions in CYP2C9 PMs (CYP2C9*2/*2, *3/*3 and *2/*3) and 25% reduction in IMs (heterozygous carriers of the *2 and *3 allele of *CYP2C9*) are recommended in guidelines.

### Drugs metabolized by CYP1A2

CYP1A2 metabolizes a small number of drugs, but three of these are included in the ISS repository: caffeine, melatonin and lidocaine. Compared to the other polymorphic enzymes, there are fewer reported variants that impact CYP1A2 activity. The genetic component of variation in CYP1A2 activity is estimated at up to 75%, while the rest is made up by environmental factors such as smoking (induction) and oral contraceptive use in women (inhibition) [57]. Variants have been described that neutralize the effect of inducers [58]. For this reason, warnings mostly concern drug-drug interactions.

### Drugs mainly eliminated by drug transporters

No warnings or information on drug transporter polymorphisms are available from the drug labels, reflecting current lack of direct evidence on exposure effects in individual drugs. P-glycoprotein, coded by the human MDR1 (ABCB1) gene, has broad substrate specificity, including most of the drugs that are metabolized by CYP3A4. The abundant expression of this efflux transporter in compartments such as the gut and the blood-brain barrier causes variability in oral absorption and central nervous drug effects. While a number of single-nucleotide polymorphisms have been identified, none causes low or absent transporter activity. However, P- glycoprotein transport activity can be largely affected by comedication with P- glycoprotein inhibiting drugs. The drugs in the ISS list that are mainly eliminated by P-glykoprotein are tobramycin, glycopyrrolate, hydromorphone and ceftriaxone.

OCT1 (Organic Cation Transporter 1), is a drug uptake transporter mainly affecting hepatic clearance of drugs. OCT1 is highly genetically variable with several loss-of-function polymorphisms affecting nine percent of Caucasians, in which there is no substantial OCT1 activity. However, no studies exist investigating the effects of OCT1 variants on drug exposure of salbutamol or ranitidine, the two drugs from the ISS list that are OCT substrates. For the remaining 4 drugs with drug transport playing a major role in elimination (moxifloxacin, ciprofloxacin, Lisinopril and amoxicillin), no data on transporter polymorphisms exist.

## Conclusion

Analysis of the drugs available on the ISS for use in human space flight revealed a similar proportion of polymorphic enzymes involved in the metabolism as is known for commonly used drugs in general (about one third) [35](Fig 1). Thus, in about every third drug, individual dose adjustments or therapy modifications may be recommended for individuals who belong to the extreme metabolizer groups such as the poor or the ultrarapid metabolizers.

Isolation from ground support may impose unprecedented demands on the health and well-being of astronauts undertaking missions to the International Space Station (ISS) or in future much longer missions out of Low Earth Orbit. Therefore, all efforts should be made to estimate and predict the individual efficacy-to-risk ratio of a particular drug therapy.

For this reason, pharmacogenetic testing during the training phase would provide information to ground crew physicians in case of pharmacological treatment in space. Many drugs commonly used in clinical practice and listed in the ISS drug supply are now labelled with pharmacogenetic information to aid physicians in choosing the most appropriate medication and in prescribing the optimal dose. This information may be used to to avoid drug failure and increase individual safety in astronauts.
